# Supplementary material for: Efficient, cell-type-specific production of flavonols by multiplexed CRISPR activation of a suite of metabolic enzymes
Source: Nat Commun. 2025 Jul 16;16:6559. doi: 10.1038/s41467-025-61742-w (PMC12267567; doi:10.1038/s41467-025-61742-w)
Supplement: Supplementary file 2 — Supplementary Information [file 41467_2025_61742_MOESM2_ESM.pdf]

Supplementary Figure 1

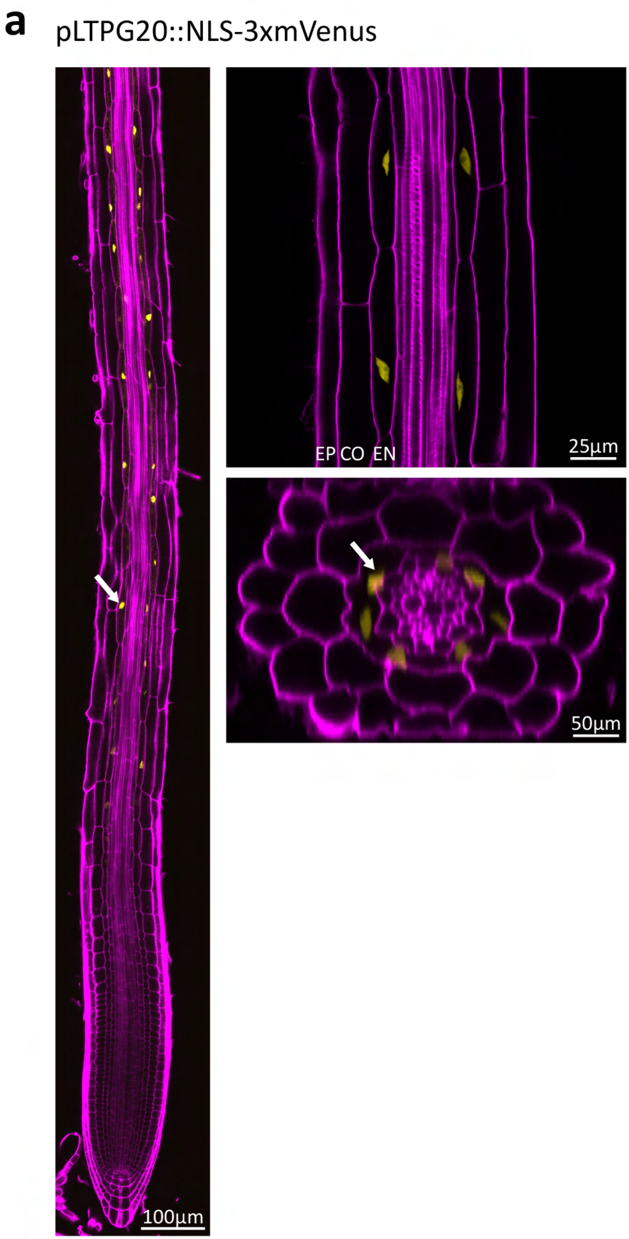

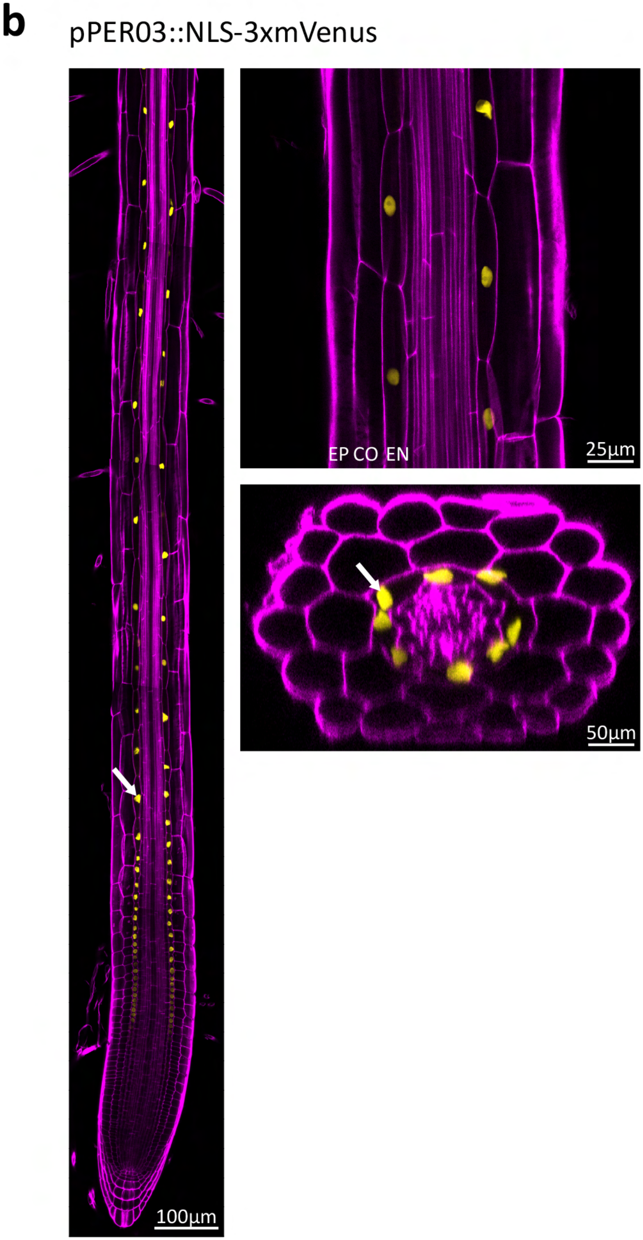

Supplementary Figure1: Transcriptional reporters of LTPG20 and PER03 in *Arabidopsis* roots

Transcriptional reporters pLTPG20::NLS-3xmVenus **(a)** and pPER03::NLS-3xmVenus **(b)** in 5-day-old roots. Seedlings were fixed with PFA and stained with CW to visualize the cell walls. LTPG20 is expressed specifically in the endodermis around endodermal differentiation. PER03 is expressed specifically in the endodermis from the division zone onwards. White Arrowheads indicate endodermis. EP epidermis, CO cortex, EN endodermis. Scale bars 100µm (root tip), 25µm (differentiated zone) and 50µm (orthogonal view).

Supplementary Figure 2

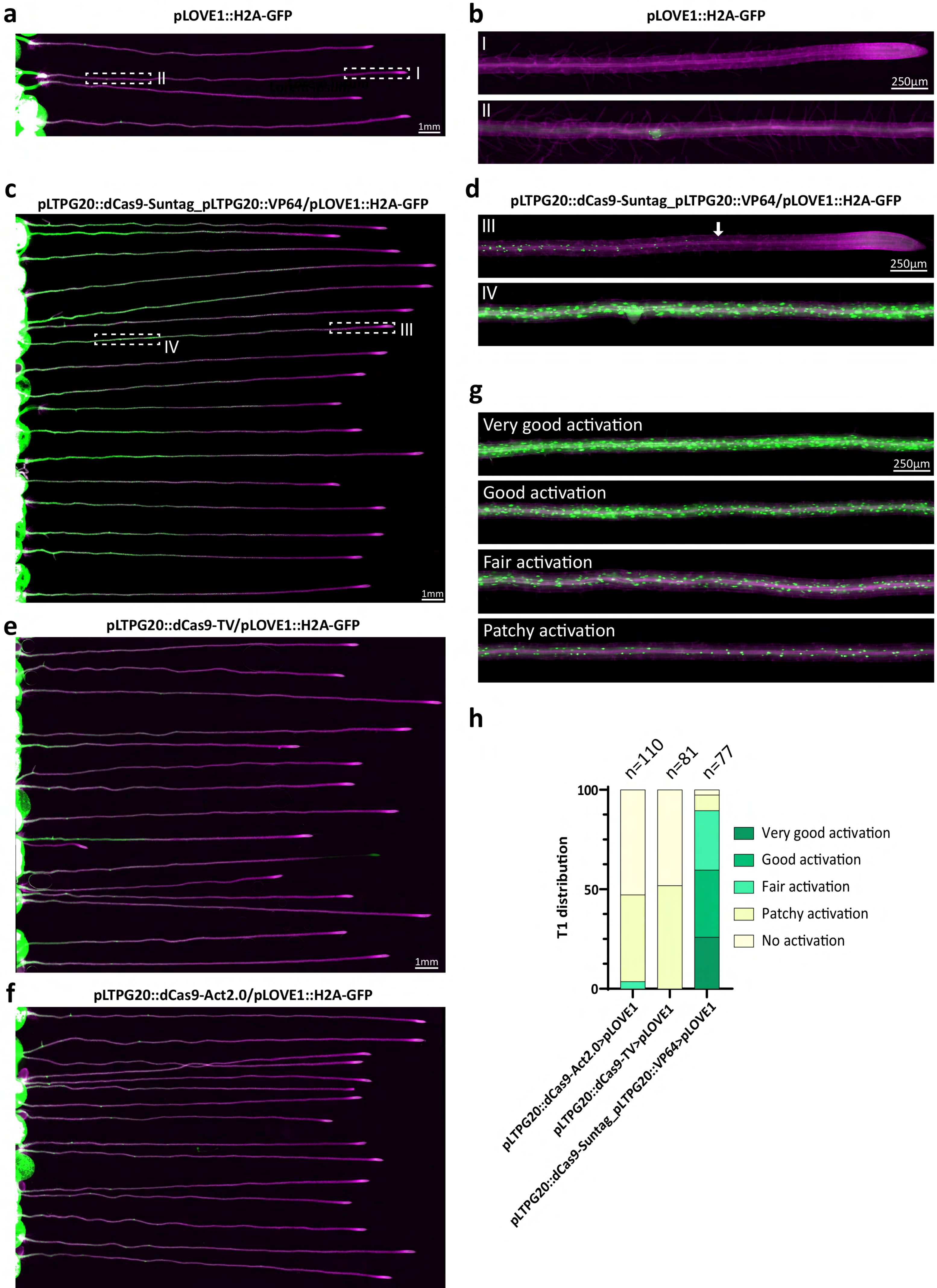

Supplementary Figure 2

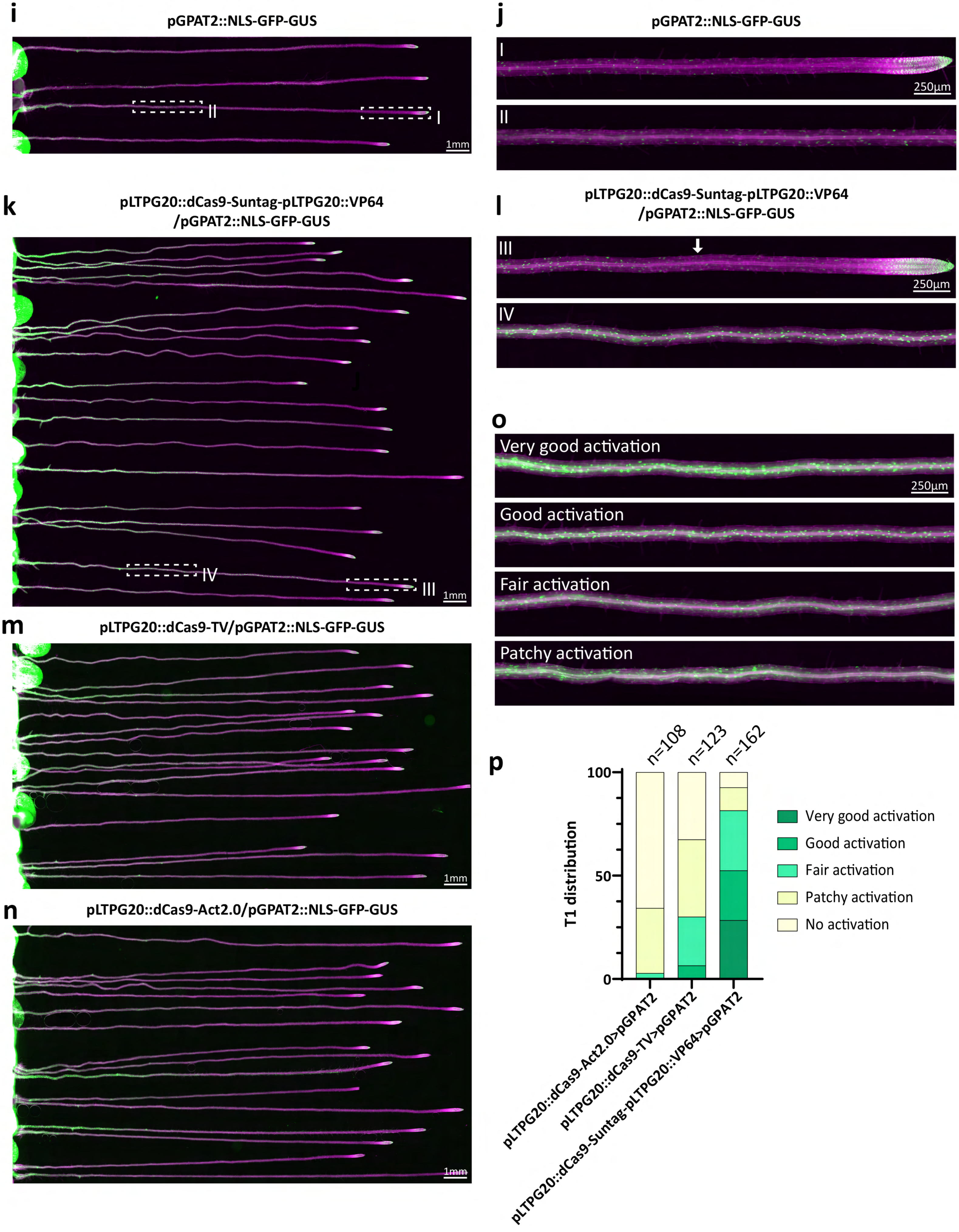

Supplementary Figure2: Comparison of the CRISPR activation systems Act2.0, TV and Suntag in roots of *Arabidopsis*

(a) 5-day-old seedlings of the pLOVE1::H2A-GFP transcriptional reporter. LOVE1 is expressed specifically in the lateral root cap of both primary (inset I) and lateral roots (inset II). Scale bar 1mm

(b) Insets shown in **(a)**. Scale bar 250µm

(c) 5-day-old seedlings of the pLOVE1::H2A-GFP reporter transformed with the dCas9-Suntag activation system expressed specifically in the endodermis. Scale bar 1mm

(d) Insets shown in **(c)**. Scale bar 250µm

(e) 5-day-old seedlings of the pLOVE1::H2A-GFP reporter transformed with the dCas9-TV activation system expressed specifically in the endodermis. Scale bar 1mm

(f) 5-day-old seedlings of the pLOVE1::H2A-GFP reporter transformed with the dCas9-Act2.0 activation system expressed specifically in the endodermis. Scale bar 1mm

(g) Representative images of the groups shown in the T1 distribution graph in **(h)**.

(h) The distribution of independent T1 lines based on their activation potential. Individual seedlings were scored based on the activation strength and stability of the pLOVE1::H2A-GFP reporter in the endodermis. The seedlings were arbitrarily attributed to one of the five groups ("Very good activation", "Good activation", "Fair activation", "Patchy or weak activation" and "No activation"). Scale bars 250µm. **(i)** 5-day-old seedlings of the pGPAT2::NLS-GFP-GUS transcriptional reporter. GPAT2 is expressed specifically in the lateral root cap and epidermis and in the cortex in more mature regions of the root. Scale bar 1mm

(j) Insets shown in **(i)**. Scale bar 250µm

(k) 5-day-old seedlings of the pGPAT2::NLS-GFP-GUS reporter transformed with the dCas9-Suntag activation system expressed specifically in the endodermis. Scale bar 1mm

(l) Insets shown in **(k)**. Scale bar 250µm

(m) 5-day-old seedlings of the pGPAT2::NLS-GFP-GUS reporter transformed with the dCas9-TV activation system expressed specifically in the endodermis. Scale bar 1mm.

(n) 5-day-old seedlings of the pGPAT2::NLS-GFP-GUS reporter transformed with the dCas9-Act2.0 activation system expressed specifically in the endodermis. Scale bar 1mm.

(o) Representative images of the groups shown in the T1 distribution graph in **(p)**.

(p) The distribution of independent T1 lines based on their activation potential. Individual seedlings were scored based on the activation strength and stability of the pGPAT2::NLS-GFP-GUS reporter in the endodermis as in **(h).** Scale bars 250µm. The seedlings in **(a-p)** were fixed in PFA and stained using calcofluor white to visualize the cell walls. n = number of independent T1 seedlings analyzed.

Supplementary Figure 3

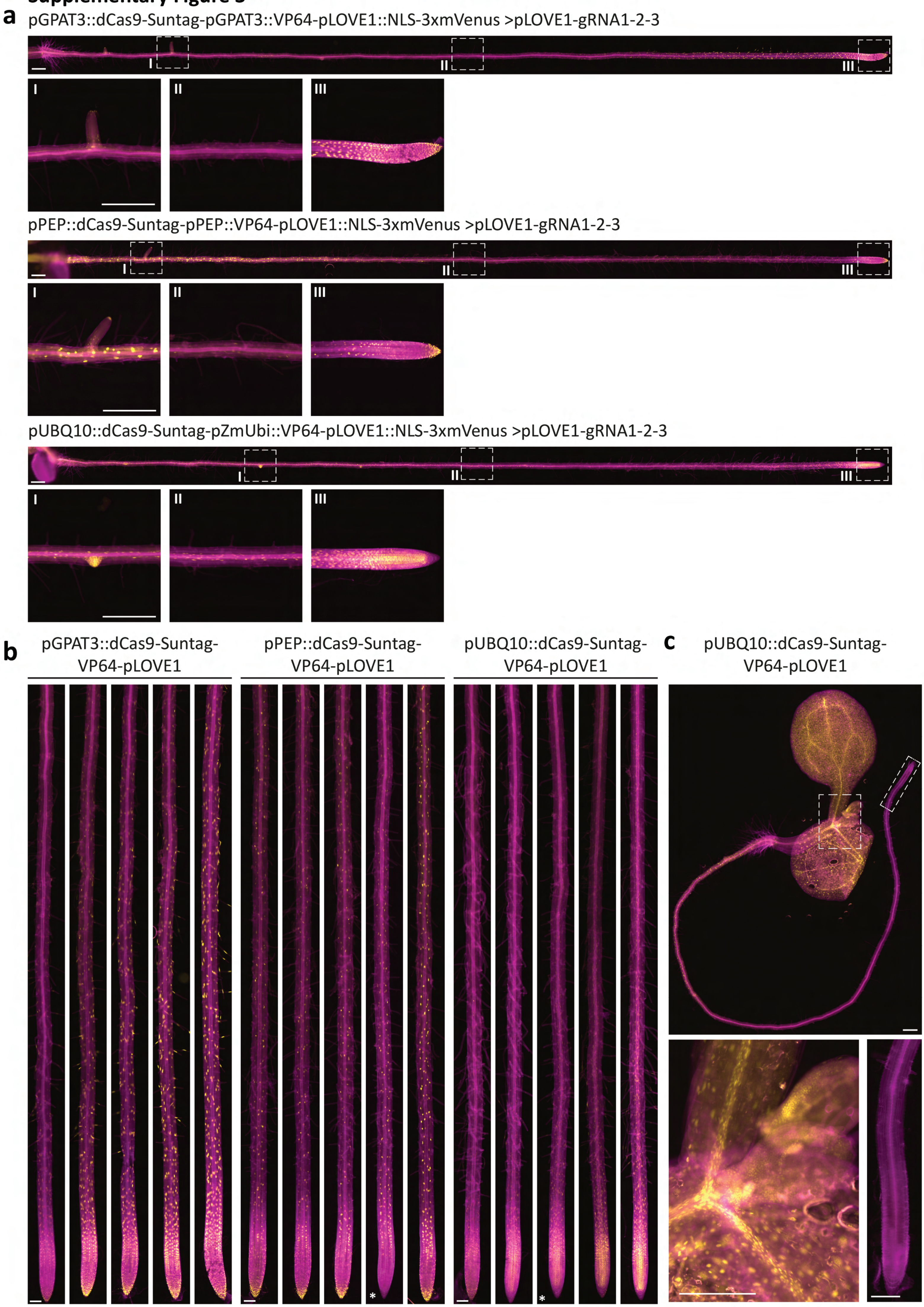

Supplementary Figure 3: Tissue specific activation in Arabidopsis seedlings using dCas9-Suntag
(a) Tissue specific transcriptional activation of the LOVE1 reporter *pLOVE1::NLS-3xmVenus* in roots of 5-day-old seedlings. Expression of the dCas9-Suntag-VP64 activation system in the epidermis and root cap using the *GPAT3* promoter (*pGPAT3::dCas9-Suntag_pGPAT3::ScFv-VP64_pLOVE1::NLS-3xmVenus >pU3/U6::pLOVE1gRNAs 1,2,3*) leads to transactivation of the *pLOVE1::NLS-3xmVenus* reporter in the root epidermis of the main root and of lateral roots. Expression of the dCas9-Suntag-VP64 activation system in the cortex using the PEP promoter (*pPEP::dCas9-Suntag_pPEP::VP64_pLOVE1::NLS-3xmVenus >pU3/U6::pLOVE1gRNAs 1,2,3*) leads to transactivation of the *pLOVE1::NLS-3xmVenus* reporter in the cortex of the main root and of lateral roots. Ubiquitous expression of the dCas9-Suntag-VP64 activation system using the UBQ10 and ZmUbi promoters (*pUBQ10::dCas9-Suntag_pZmUbi::ScFV-VP64-pLOVE1::NLS-3xmVenus > pU3/U6::pLOVE1gRNAs 1,2,3*) leads to transactivation of the pLOVE1::NLS-3xmVenus reporter in all tissue types of the main root and of lateral roots. Insets are shown below the full root. Scale bars = 250µm
(b) Representative independent T1 lines presented in **(a)**. Asterisks represent silencing of the *pLOVE1::NLS-3xmVenus* reporter. Scale bars = 100µm
(c) Seedling expressing the pUBQ10::dCas9-Suntag-VP64 activation system. The expression of the *pLOVE1::NLS-3xmVenus* reporter is activated in all cell types of the shoot whilst its expression is silenced in the root. Scale bars = 250µm

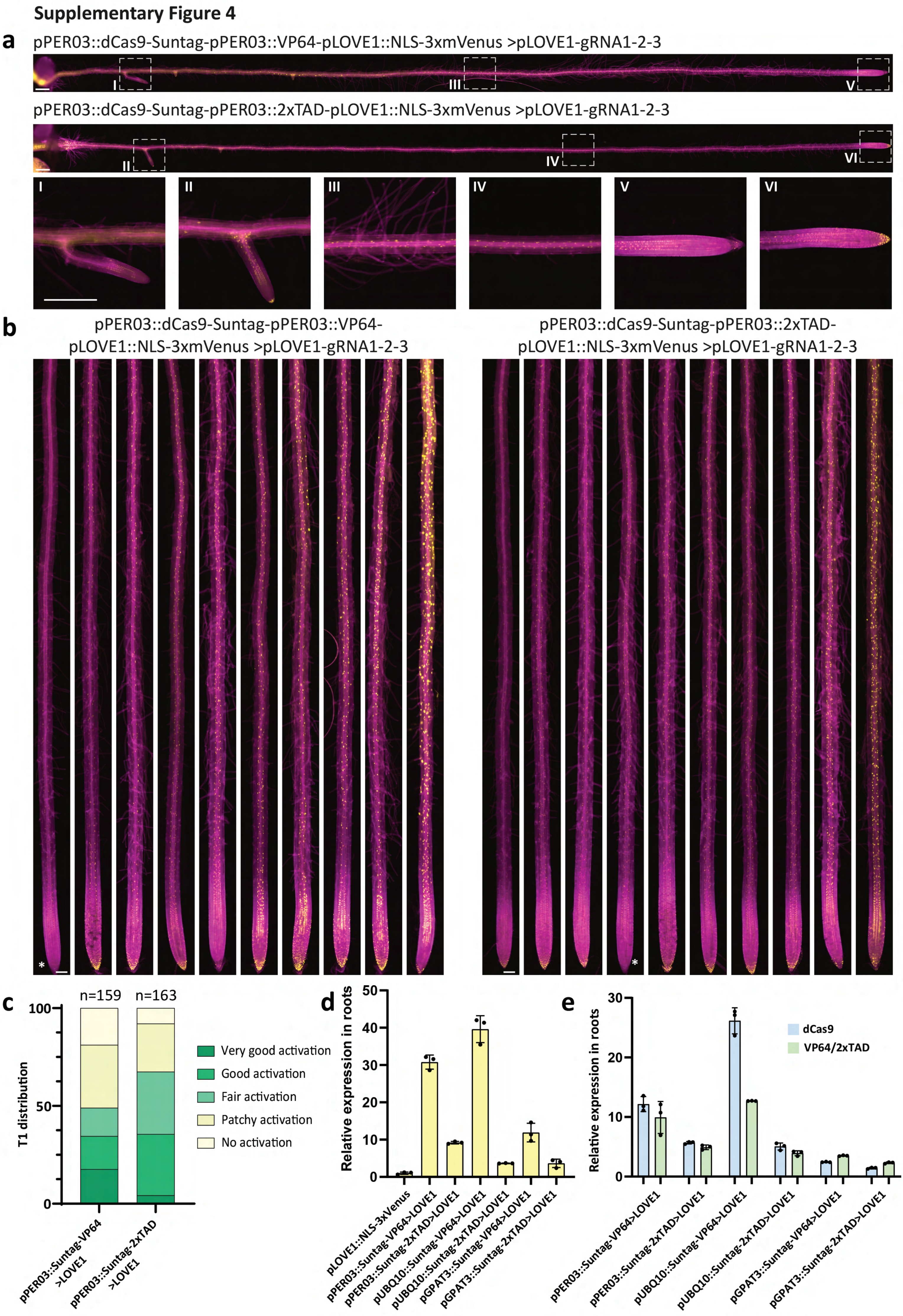
Supplementary Figure 4
a
pPER03::dCas9-Suntag-pPER03::VP64-pLOVE1::NLS-3xmVenus >pLOVE1-gRNA1-2-3
pPER03::dCas9-Suntag-pPER03::2xTAD-pLOVE1::NLS-3xmVenus >pLOVE1-gRNA1-2-3
I
II
III
IV
V
VI
b
pPER03::dCas9-Suntag-pPER03::VP64-pLOVE1::NLS-3xmVenus >pLOVE1-gRNA1-2-3
pPER03::dCas9-Suntag-pPER03::2xTAD-pLOVE1::NLS-3xmVenus >pLOVE1-gRNA1-2-3
c
n=159
n=163
T1 distribution
100
50
0
Very good activation
Good activation
Fair activation
Patchy activation
No activation
pPER03::Suntag-VP64 >LOVE1
pPER03::Suntag-2xTAD >LOVE1
d
Relative expression in roots
50
40
30
20
10
0
pLOVE1::NLS-3xVenus
pPER03::Suntag-VP64>LOVE1
pPER03::Suntag-2xTAD>LOVE1
pUBQ10::Suntag-VP64>LOVE1
pUBQ10::Suntag-2xTAD>LOVE1
pGPAT3::Suntag-VP64>LOVE1
pGPAT3::Suntag-2xTAD>LOVE1
e
Relative expression in roots
30
20
10
0
dCas9
VP64/2xTAD
pPER03::Suntag-VP64>LOVE1
pPER03::Suntag-2xTAD>LOVE1
pUBQ10::Suntag-VP64>LOVE1
pUBQ10::Suntag-2xTAD>LOVE1
pGPAT3::Suntag-VP64>LOVE1
pGPAT3::Suntag-2xTAD>LOVE1

Supplementary Figure 4

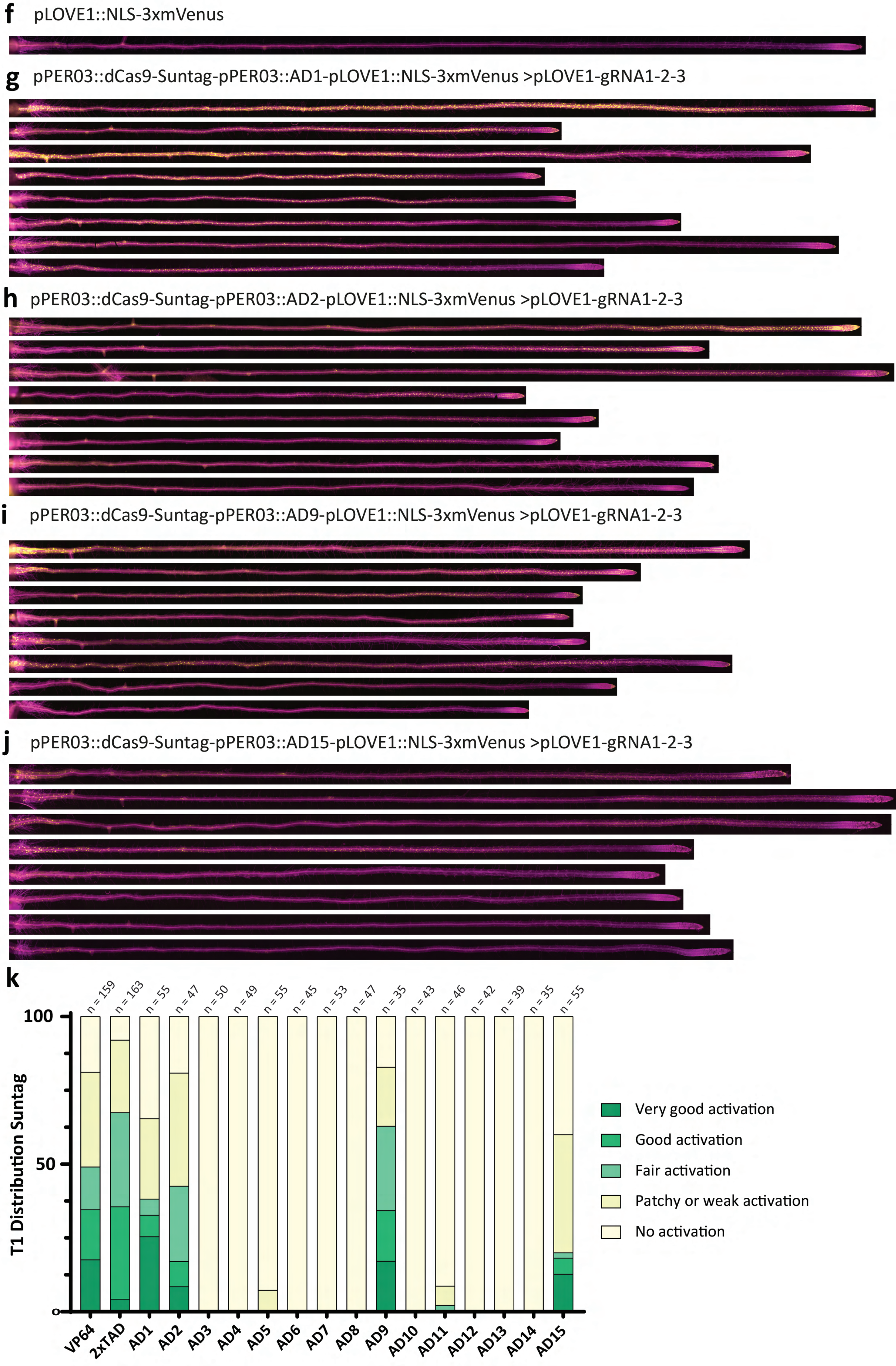

Supplementary Figure 4: VP64 has a stronger activation potential than 2xTAD

(a) Endodermis specific transcriptional activation of the *pLOVE1::NLS-3xmVenus* reporter in roots of 5-day-old seedlings. Expression of the dCas9-Suntag-VP64 or dCas9-Suntag-2xTAD activation system in the endodermis using the PER03 promoter leads to transactivation of the LOVE1 reporter in the endodermis of the main root and of lateral roots. Insets are shown below the full root. Scale bars = 250µm

(b) Representative independent VP64 or 2xTAD T1 lines. VP64 on average shows a stronger activation than 2xTAD but the latter is more consistent. Asterisks represent silencing of the *pLOVE1::NLS-3xmVenus* reporter. Scale bars = 100µm

(c) The distribution of independent T1 lines. Individual seedlings were scored based on the activation strength and stability of the *pLOVE1::NLS-3xmVenus* reporter in the endodermis. The seedlings were arbitrarily attributed to one of the five groups ("Very good activation", "Good activation", "Fair activation", "Patchy or weak activation" and "No activation"). n = individual seedlings analyzed.

(d) Relative expression of 3xmVenus in roots of 150 pooled independent 5-day-old seedlings expressing the VP64 or 2xTAD activation systems under the promoters PER03 (endodermis), UBQ10 (all tissues) or GPAT3 (root epidermis and root cap). VP64 shows on average a stronger activation potential of the *pLOVE1::NLS-3xmVenus* reporter. The graph displays a representative experiment from 3 independent biological repeats. Data are presented as mean values, and the error bars indicate the standard deviations (SD).

(e) Relative expression of the VP64 or 2xTAD systems in roots 5-day-old seedlings shown in **(d)**. Both dCas9-Suntag and ScFv-VP64 transcripts show on average a stronger expression in VP64 lines than in 2xTAD lines.

(f) Root cap specific expression of the LOVE1 reporter *pLOVE1::NLS-3xmVenus* in roots of 5-day-old seedlings.

(g) Endodermis specific transcriptional activation of the LOVE1 reporter *pLOVE1::NLS-3xmVenus* in roots of 5-day-old seedlings expressing the dCas9-Suntag-AD1 **(g)**, dCas9-Suntag-AD2 **(h),** dCas9-Suntag-AD9 **(i)** or dCas9-Suntag-AD15 **(j)** activation systems expressed under the PER03 promoter. Scale bars in **(g-j)** = 250µm

(k) The distribution of independent T1 lines. Individual seedlings were scored based on the activation strength and stability of the *pLOVE1::NLS-3xmVenus* reporter in the endodermis as in **(c)**.

Supplementary Figure 5

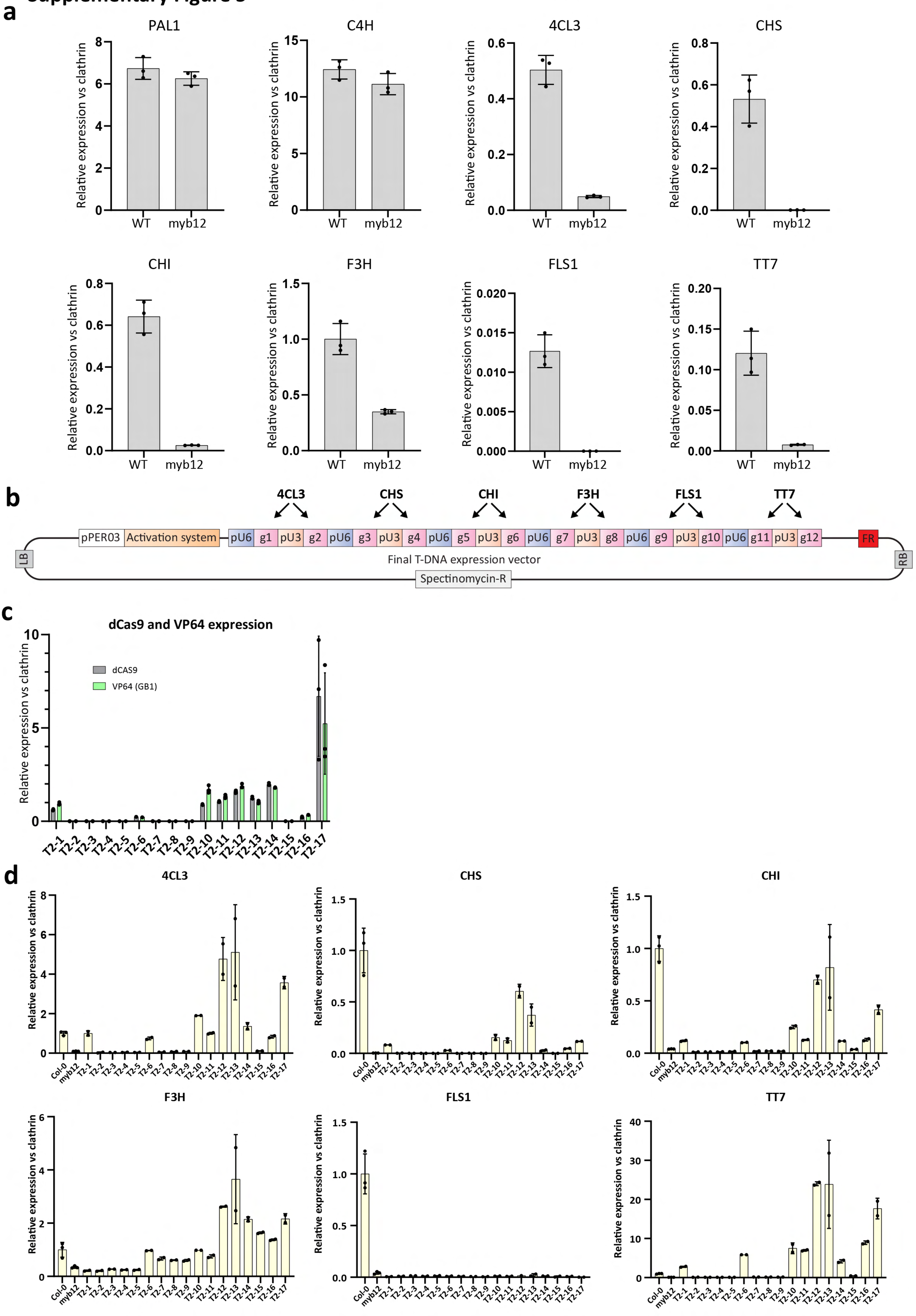

Supplementary Figure 5: Expression of flavonol biosynthetic genes in myb12 and activation lines

(a) Relative expression levels (vs clathrin) of PAL1, C4H, 4CL3, CHS, CHI, F3H, FLS1 and TT7 in roots of 5-day-old seedlings of Col-0 and *myb12* measured by qPCR.

(b) Schematic representation of the expression vector harboring the *pPER03::dCas9-Suntag_pPER03::ScFv-VP64* activation system and 12gRNAs targeting 4CL3, CHS, CHI, F3H, FLS1 and TT7 (2gRNAs each).

(c) Relative expression levels (vs clathrin) of 4CL3, CHS, CHI, F3H, FLS1 and TT7 in roots of 5-days-old seedlings of independent T2 lines in *myb12* coexpressing the Suntag activation system under control of the PER03 promoter and 2 gRNAs targeting 4CL3, CHS, CHI, F3H, FLS1 and TT7. About 30% of the lines show transcriptional activation for 4CL3, CHS, CHI, F3H, FLS1 and TT7. FLS1 could not be activated with gRNA1 and gRNA2.

(d) Relative expression levels of dCas9 or VP64 activation domain in the independent T2 lines presented in **(a)**.

Supplementary Figure 6

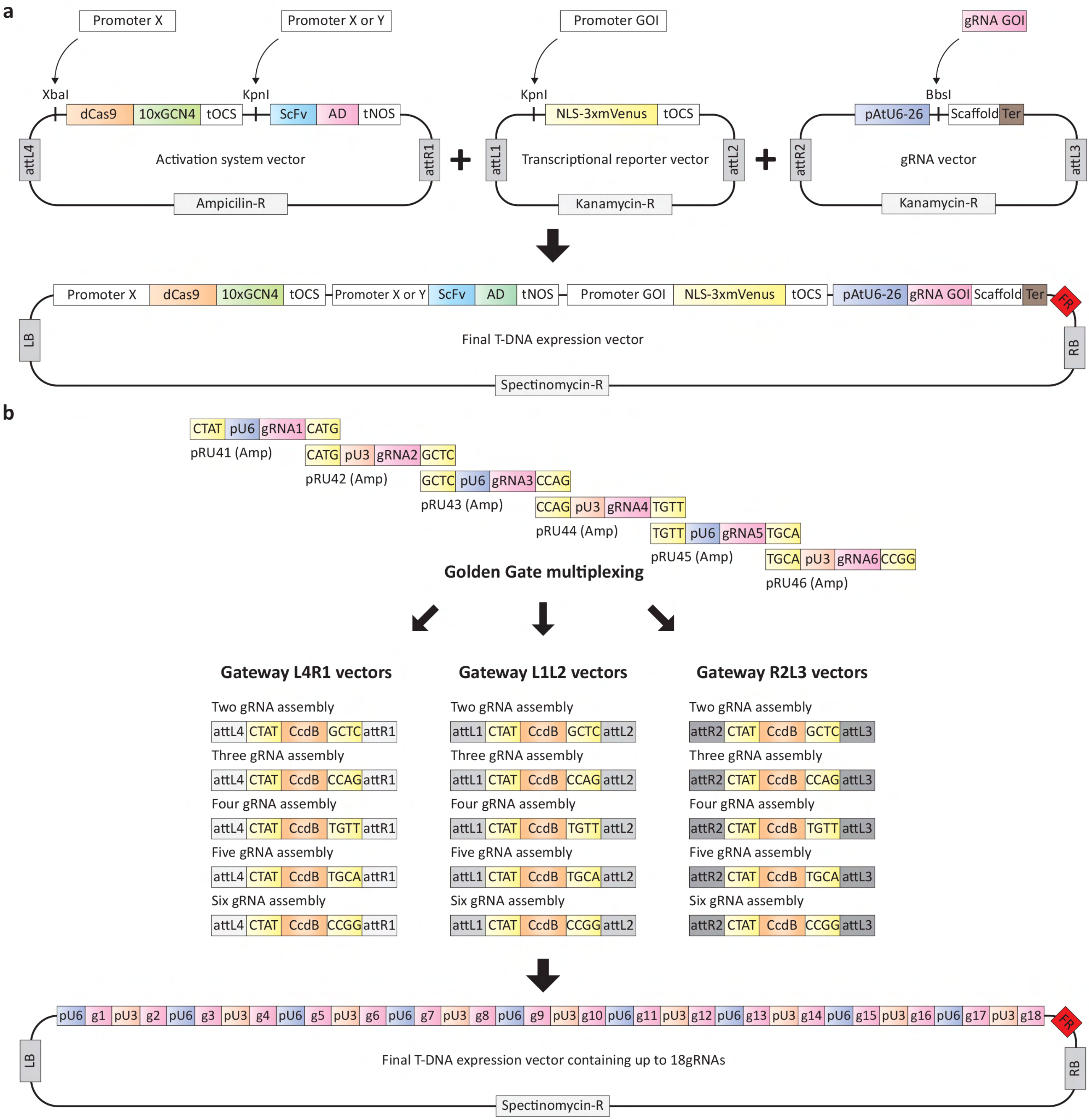

Supplementary Figure 6: Cloning strategy for generating gateway entry vectors

(a) Schematic representation of the cloning strategy for gateway compatible entry vectors conta ining the activation system (left), the transcriptional reporter (middle) and the gRNA expression cassette (right) used for testing gRNA activation potential. Clone the promoter X in front of the dCas9-10xGCN4 cassette using XbaI and the promoter X or Y in front of the ScFv-AD (activation domain) using KpnI to generate a cell type specific activation system (Gateway entry clone L4R1). Clone the promoter of the gene of interest (GOI) in front of the NLS-3xmVenus-tOCS cassette using KpnI to generate a transcriptional reporter (Gateway entry clone L1L2). Clone a single gRNA targeting the promoter region of the gene of interest into an gateway entry clone R2L3 using BbsI and oligo annealing. The entry clones can be recombined using an LR reaction into a destination vector containing a red fluorescent (FR) seed selection marker (or any other compatible vector). Following Arabidopsis transformation, around 20 red fluorescent seeds can be selected to assess the gRNA activation potential in T1 seedlings.

(b) Schematic representation showing the multiplexing strategy for stacking gRNA cassettes into T-DNA expression vectors. Single gRNAs are cloned into intermediate vectors containing either a U6 or a U3 promoter, respectively, and a terminator (pRU41, pRU42, pRU43, pRU44, pRU45 and pRU46). Using Golden gate, gRNAs are stacked into gateway compatible entry vectors (position 1: L4R1, position 2: L1L2 and position 3 R2L3). The obtained entry clones are recombined into a single destination vector allowing the expression of up to 18gRNAs.

Supplementary Figure 7

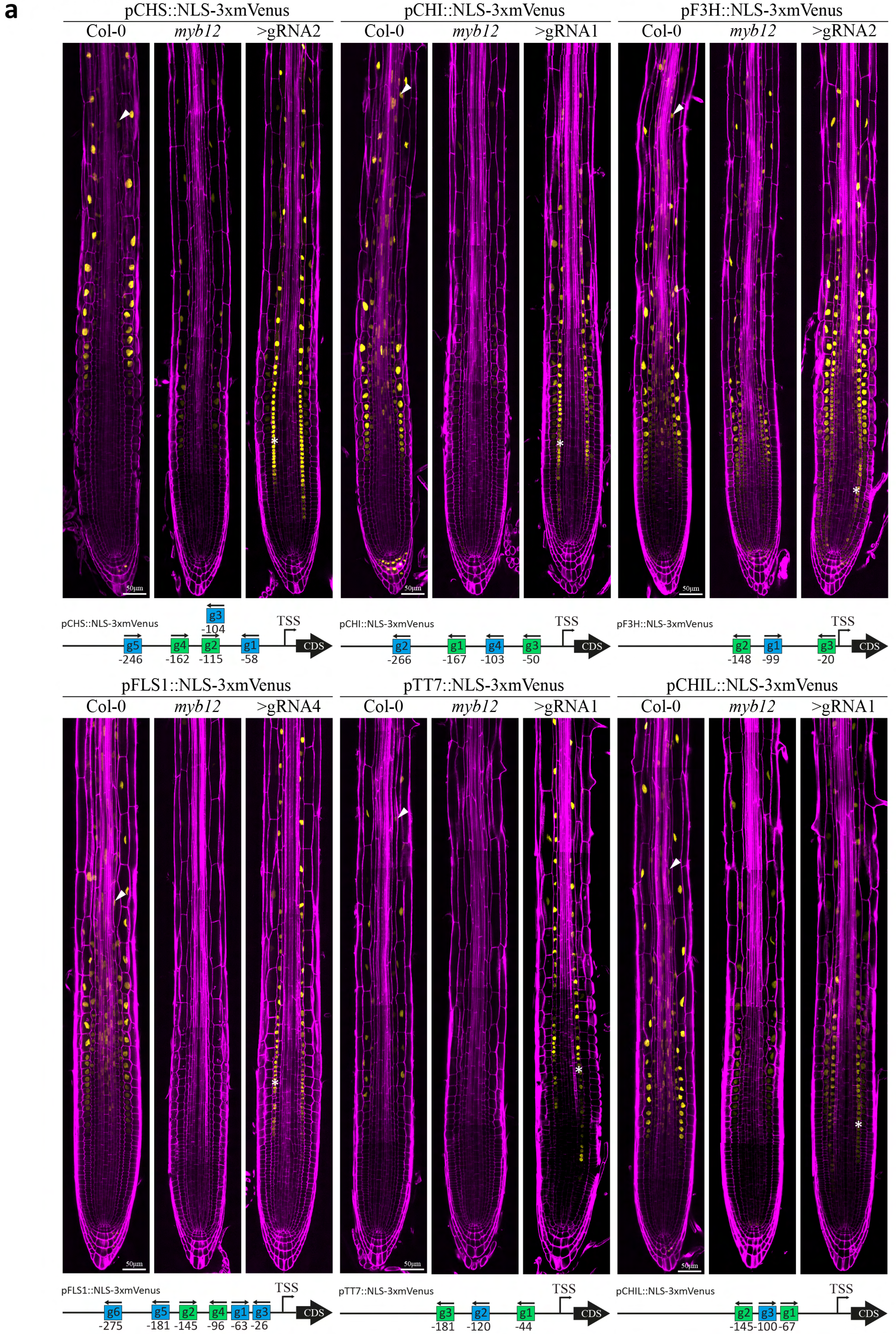

Supplementary Figure 7

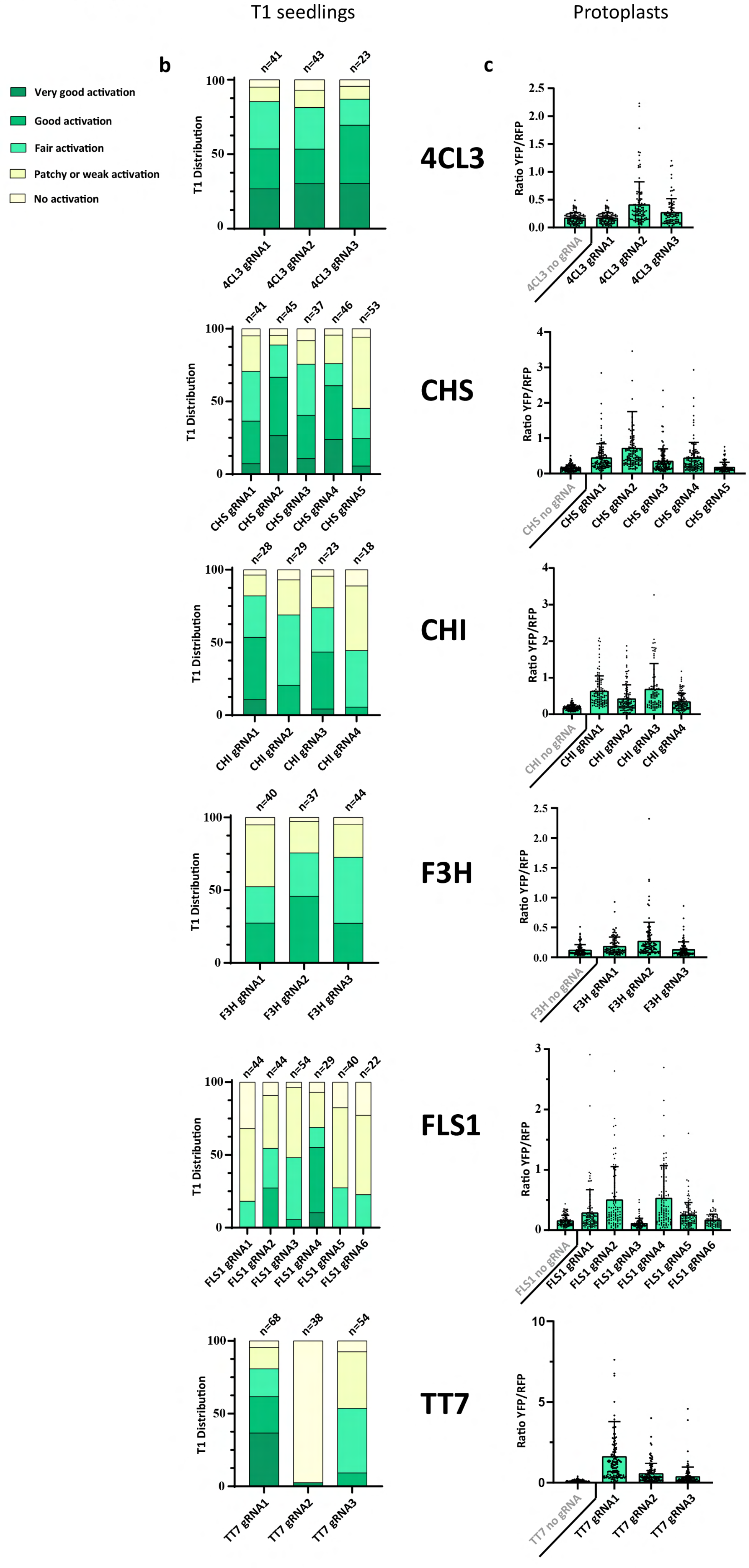

Supplementary Figure 7: Testing of individual gRNA efficiency for activation of flavonol biosynthetic enzymes in stable T1 lines or protoplasts

(a) Transcriptional reporters of pCHS::NLS-3xmVenus, pCHI::NLS-3xmVenus, pF3H::NLS-3xmVenus, pFLS1::NLS-3xmVenus, pTT7::NLS-3xmVenus and pCHIL::NLS-3xmVenus in Col-o and *myb12*. CHS, CHI, F3H, FLS1, TT7 and CHIL are expressed in the endodermis in Col-0 (white arrowheads) but are no longer or barely expressed there in *myb12,* except for F3H. gRNA activation potential in the endodermis of *myb12* was scored by expressing the dCas9-Suntag-2xTAD activation system in the endodermis using the PER03 promoter. The gRNA positions are represented on the schematics under the picture. gRNAs in green represent gRNAs with the best activation potential. The best working gRNA was selected for imaging. Transcriptional activation in the endodermis is indicated with a white asterisk. Seedlings were fixed with PFA and stained with CW to visualize the cell walls. Scale bars 50µm. **(b)** Comparison between activation of the transcriptional reporters for 4CL3, CHS, CHI, F3H, FLS1 and TT7 in independent stable T1 lines and in protoplasts. The distribution of individual T1 seedlings were scored based on the activation strength and stability of the corresponding reporter in the root endodermis. The seedlings were arbitrarily attributed to one of the five groups (“Very good activation”, “Good activation”, “Fair activation”, “Patchy or weak activation” and “No activation”). n = number of seedlings analyzed. **(c)** For assessment of gRNA efficiency in protoplasts, the ratio was determined between the YFP signal (transcriptional reporters for 4CL3, CHS, CHI, F3H, FLS1 and TT7) and that of RFP (pUBQ10::NLS-tdTomato) allowing for a ratiometric quantification.

Supplementary Figure 8

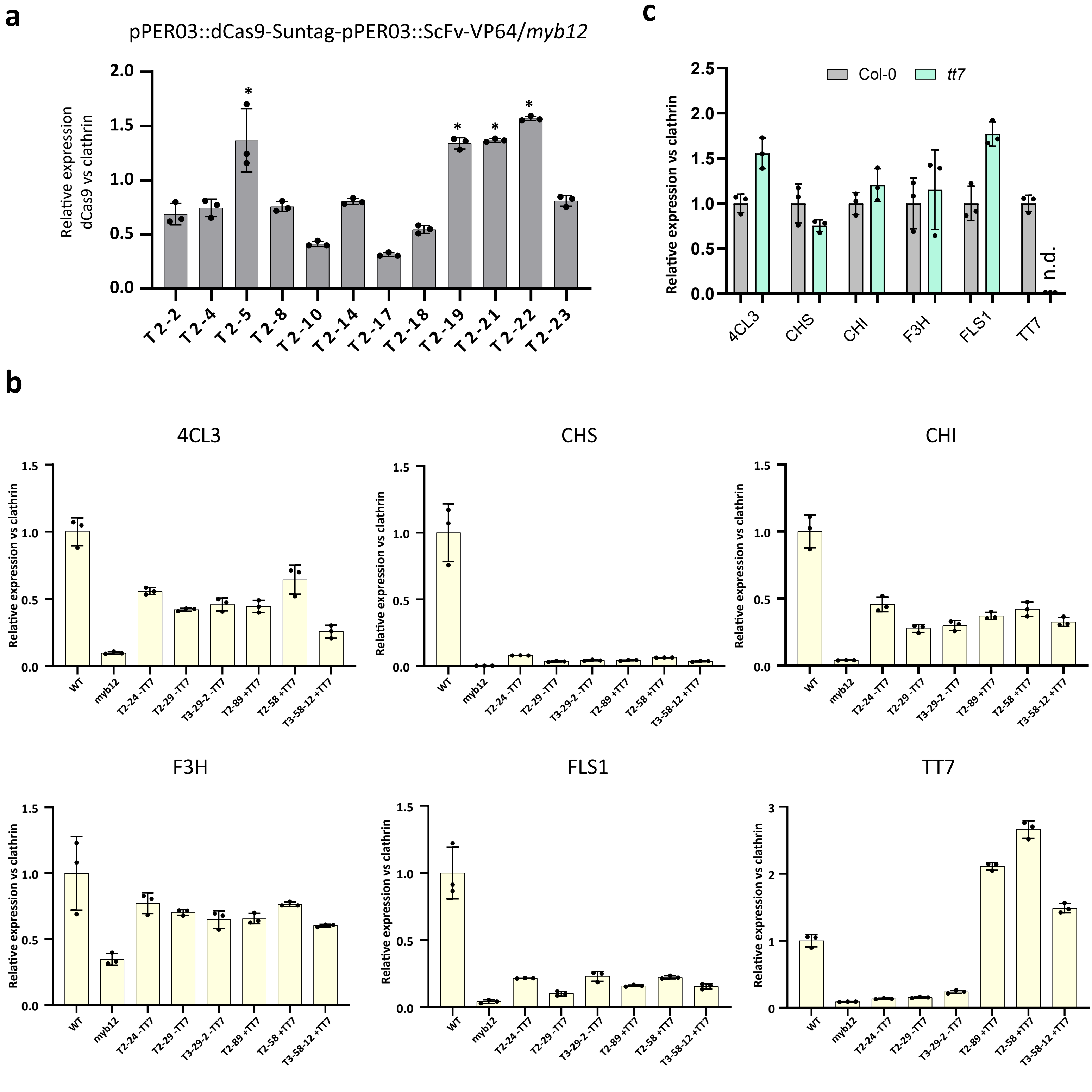

Supplementary Figure 8: Pre-selection of Cas9 lines and performance of pre-selected lines
(a) Relative expression levels of dCas9 in the independent T2 lines expressing the Suntag activation system in *myb12* under control of the PER03 promoter. The asterisk indicates lines taken to T3 to establish stable homozygous lines.
(b) Relative expression of 4CL3, CHS, CHI, F3H, FLS1 and TT7 in roots of 5-day-old seedlings of Col-0, *myb12,* two independent lines producing kaempferol (-TT7) by coactivation of 4CL3, CHS, CHI, F3H and FLS1 and two independent lines producing both kaempferol and quercetin (+TT7) by coactivation of 4CL3, CHS, CHI, F3H, FLS1 and TT7. Expression of the flavonol biosynthetic enzymes could be reconstituted in *myb12* by using the dCas9-Suntag-VP64 activation system.
(c) Relative expression of 4CL3, CHS, CHI, F3H, FLS1 and TT7 in roots of 5-day-old seedlings of Col-0 and *tt7.* The *tt7* mutant does not produce TT7 transcripts.

Supplementary Figure 9

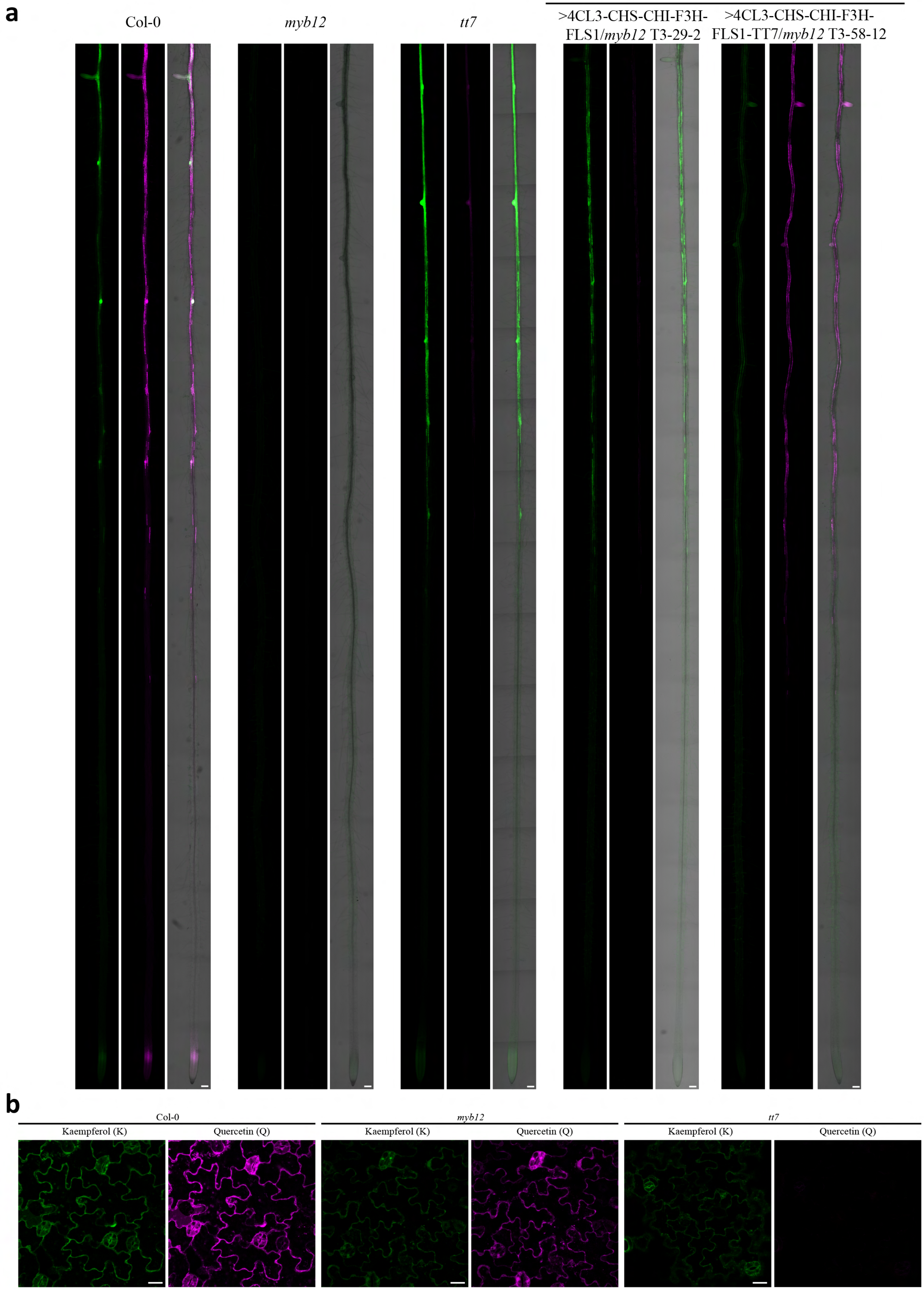

Supplementary Figure 9

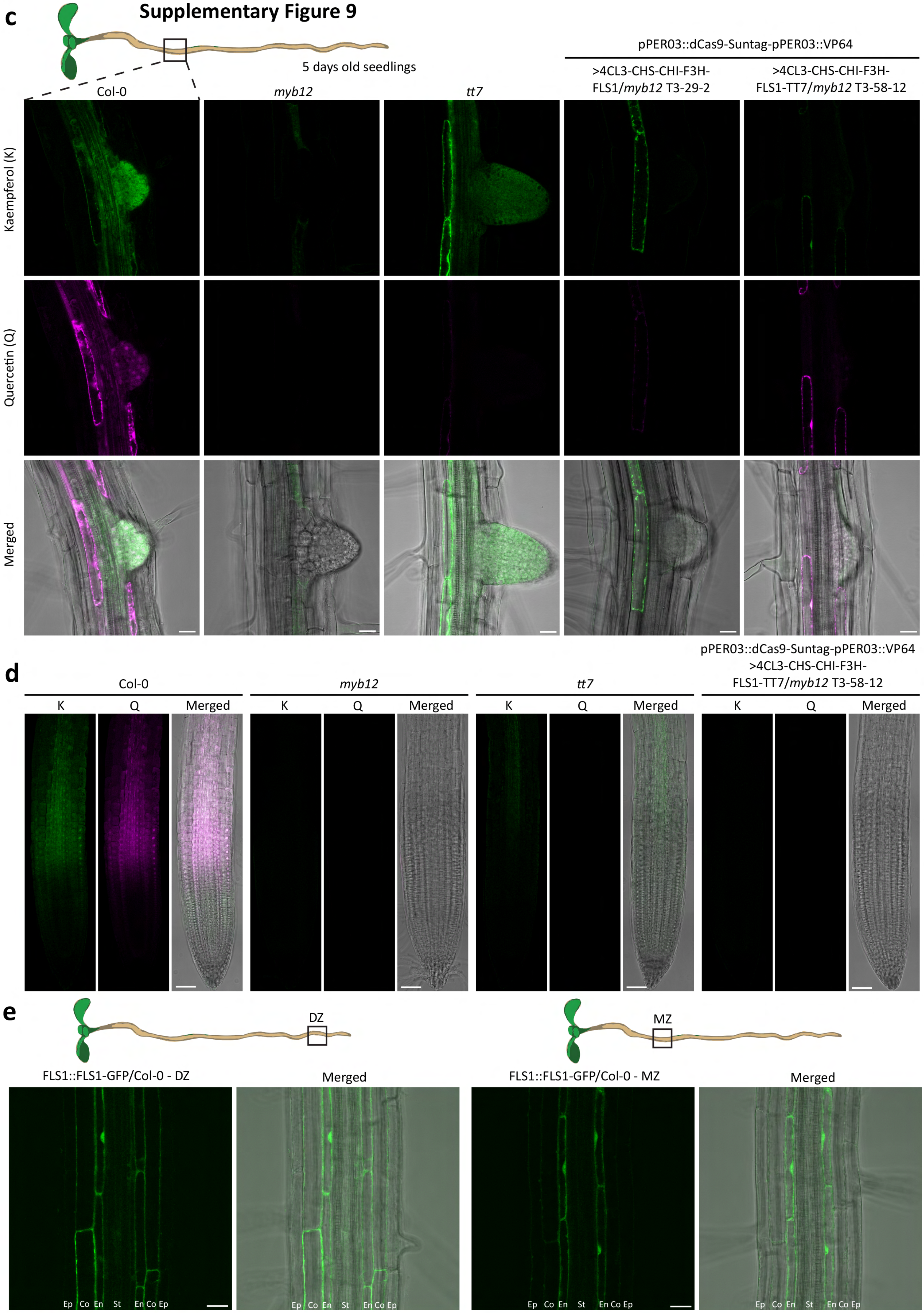

Supplementary Figure 9

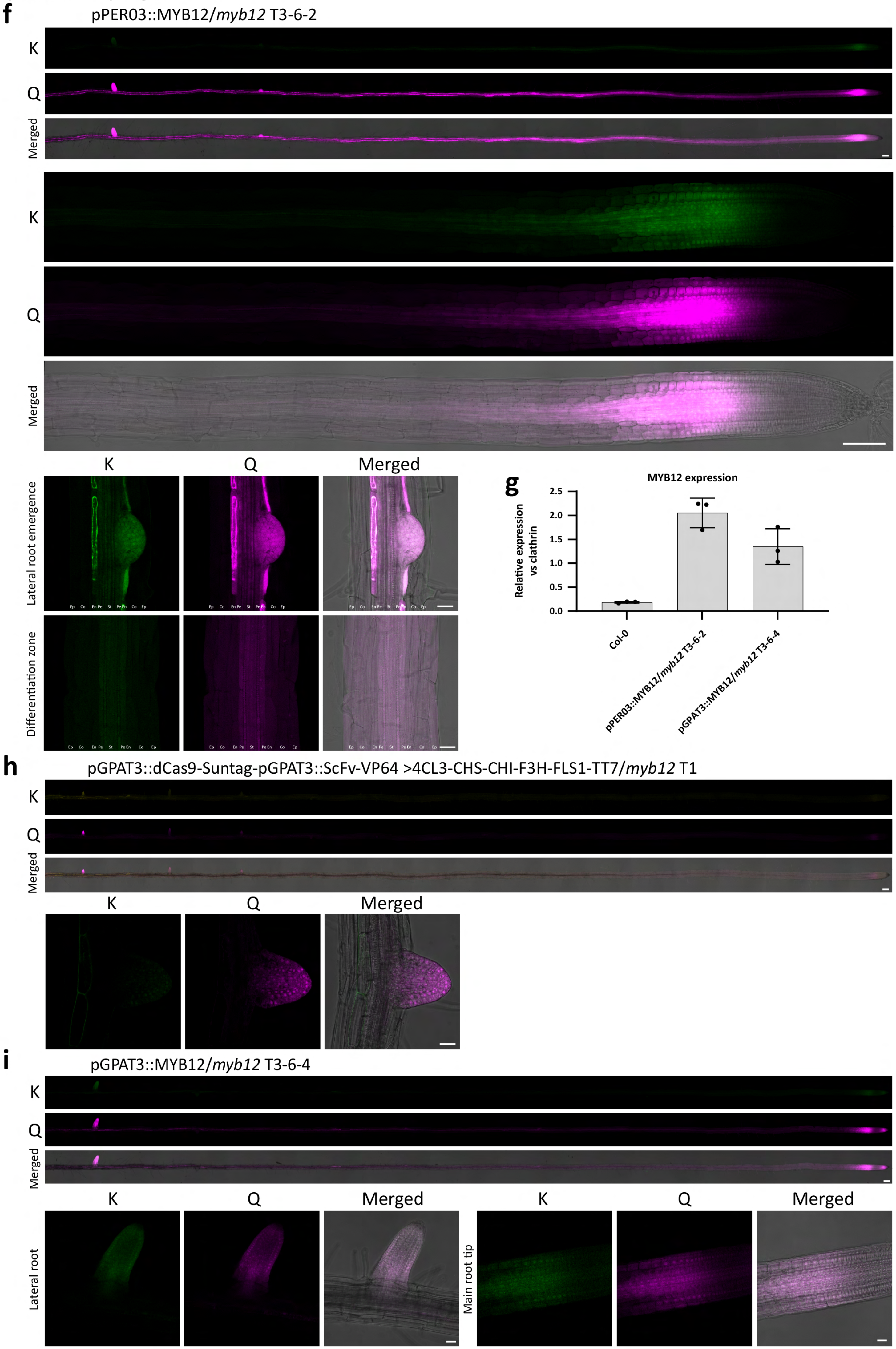

Supplementary Figure 9: Spatial flavonol accumulation by patterns by DPBA stain in different genotypes

(a) DPBA stain in roots of 5-day-old seedlings. Production of kaempferol (K) and quercetin (Q) was reconstituted specifically in the *myb12* endodermis in roots by co-activation of 4CL3, CHS, CHI, F3H, FLS1 and TT7. Co-activation without TT7 leads to production of just kaempferol. The *tt7* mutant devoid of quercetin and over-accumulating kaempferol was used as a control. Scale bars = 250µm

(b) DPBA stain in cotyledons of 5-day-old seedlings. The mutant *myb12* still produces kaempferol and quercetin in shoots. Scale bars 50µm.

(c) Close-up of lateral root regions after DPBA stain in roots of 5-day-old seedlings. In Col-0, K and Q are found in the vasculature, the pericycle and endodermis. A very low amount of K can be identified in endodermal cells surrounding developing lateral roots in the *myb12* mutant. Production of kaempferol and quercetin was reconstituted specifically in the *myb12* endodermis in roots by co-activation of 4CL3, CHS, CHI, F3H, FLS1 and TT7. Co-activation without TT7 leads to production of just kaempferol. Scale bars = 25µm

(d) Close-up of the main root tip after DPBA stain in 5-day-old seedlings. Scale bars 50µm.

(e) Translational reporter pFLS1::FLS1-GFP in the root endodermis of 5-day-old seedlings. MZ and DZ stand for mature zone and differentiated zone, respectively. Ep Epidermis, Co Cortex, En Endodermis, St Stele. Scale bars = 25µm

(f) DPBA stain in roots of 5-day-old seedlings of a stable T3 line overexpressing MYB12 under the PER03 promoter in the *myb12* mutant background. Expression of MYB12 solely in the endodermis rescues accumulation of kaempferol and quercetin in the pericycle and stele and main and lateral root meristem similar to wild type. A delay in flavonol accumulation can still be observed in the root endodermis compared to PER03 promoter activity. Scale bars: top and middle 100 µm, bottom = 25µm

(g) Relative expression levels of MYB12 in T3 lines overexpressing MYB12 under the PER03 and the GPAT3 promoter, respectively.

(h) DPBA stain in roots of 5-day-old seedlings of a stable T1 line expressing the Suntag activation system specifically in the root epidermis and root cap cells under the GPAT3 promoter. Production of kaempferol and quercetin was reconstituted in *myb12* main root (faintly) and lateral root meristems (strongly) by co-activation of 4CL3, CHS, CHI, F3H, FLS1 and TT7. Scale bars: top 100 µm, bottom = 25µm

(i) DPBA stain in roots of 5-day-old seedlings of a stable T3 line overexpressing MYB12 under the GPAT3 promoter in the *myb12* mutant background. Expression of MYB12 solely in the epidermal and root cap cells rescues accumulation of kaempferol and quercetin in the pericycle and stele (faintly) and main and lateral root meristem (strongly). Scale bars: top 100 µm, bottom = 25µm
