# Supplementary material for: Efficient, cell-type-specific production of flavonols by multiplexed CRISPR activation of a suite of metabolic enzymes
Source: Nat Commun. 2025 Jul 16;16:6559. doi: 10.1038/s41467-025-61742-w (PMC12267567; doi:10.1038/s41467-025-61742-w)
Supplement: Supplementary file 3 — Description of additional supplementary files [file 41467_2025_61742_MOESM3_ESM.pdf]

Description of Additional Supplementary Files

Supplementary Data 1: Alternative Activation Domains

This file contains the gene codes, the protein sequences and the activation score of the 15 activation domains described in Supplementary Fig. 4.

Supplementary Data 2: Primer Sequences

This file contains all the primer sequences used in this study.
